# *N*-[(*E*)-1,3-Benzodioxol-5-yl­methyl­idene]-3,4-dimethyl-1,2-oxazol-5-amine

**DOI:** 10.1107/S1600536811030327

**Published:** 2011-08-11

**Authors:** Abdullah M. Asiri, Salman A. Khan, M. Nawaz Tahir

**Affiliations:** a Department of Chemistry, Faculty of Science, King Abduaziz University, Jeddah 21589, PO Box 80203, Saudi Arabia; b University of Sargodha, Department of Physics, Sargodha, Pakistan

## Abstract

In the title compound, C_13_H_12_N_2_O_3_, the dihedral angle between the aromatic rings is 7.94 (12)°. In the crystal, inversion dimers linked by pairs of C—H⋯O hydrogen bonds generate *R*_2_^2^(6) loops. Weak π–π [centroid–centroid separations = 3.7480 (13) and 3.9047 (13) Å] and C—H⋯π inter­actions help to consolidate the packing.

## Related literature

For background to conjugated azo-methanes, see: Asiri & Khan (2010 ▶). For related structures, see: Asiri *et al.* (2010 ▶); Tahir *et al.* (2010 ▶). For graph-set notation, see: Bernstein *et al.* (1995 ▶).
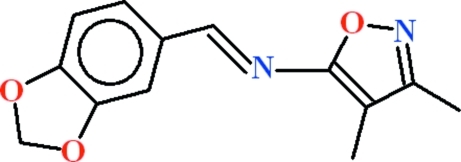


## Experimental

### 

#### Crystal data

C_13_H_12_N_2_O_3_
*M* *_r_* = 244.25
Monoclinic, 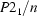

*a* = 7.5759 (5) Å
*b* = 10.6980 (9) Å
*c* = 14.6307 (12) Å
β = 102.607 (2)°
*V* = 1157.19 (16) Å^3^
*Z* = 4
Mo *K*α radiation
μ = 0.10 mm^−1^
*T* = 296 K
0.28 × 0.24 × 0.22 mm

#### Data collection

Bruker Kappa APEXII CCD diffractometer
Absorption correction: multi-scan (*SADABS*; Bruker, 2005 ▶) *T*_min_ = 0.975, *T*_max_ = 0.980
8018 measured reflections
2087 independent reflections
1447 reflections with *I* > 2σ(*I*)
*R*_int_ = 0.032

#### Refinement

*R*[*F* ^2^ > 2σ(*F* ^2^)] = 0.045
*wR*(*F* ^2^) = 0.132
*S* = 1.03
2087 reflections
165 parameters
H-atom parameters constrained
Δρ_max_ = 0.17 e Å^−3^
Δρ_min_ = −0.18 e Å^−3^

### 

Data collection: *APEX2* (Bruker, 2009 ▶); cell refinement: *SAINT* (Bruker, 2009 ▶); data reduction: *SAINT*; program(s) used to solve structure: *SHELXS97* (Sheldrick, 2008 ▶); program(s) used to refine structure: *SHELXL97* (Sheldrick, 2008 ▶); molecular graphics: *ORTEP-3 for Windows* (Farrugia, 1997 ▶) and *PLATON* (Spek, 2009 ▶); software used to prepare material for publication: *WinGX* (Farrugia, 1999 ▶) and *PLATON*.

## Supplementary Material

Crystal structure: contains datablock(s) global, I. DOI: 10.1107/S1600536811030327/hb6330sup1.cif

Structure factors: contains datablock(s) I. DOI: 10.1107/S1600536811030327/hb6330Isup2.hkl

Supplementary material file. DOI: 10.1107/S1600536811030327/hb6330Isup3.cml

Additional supplementary materials:  crystallographic information; 3D view; checkCIF report

## Figures and Tables

**Table 1:** Hydrogen-bond geometry (Å, °) *Cg*1 is the centroid of the C1–C6 benzene ring.

*D*—H⋯*A*	*D*—H	H⋯*A*	*D*⋯*A*	*D*—H⋯*A*
C7—H7*B*⋯O1^i^	0.97	2.58	3.264 (3)	128
C12—H12*A*⋯*Cg*1^ii^	0.96	2.95	3.763 (2)	143

Symmetry codes: (i) 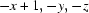; (ii) 
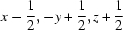
.

**Fig. 1.**
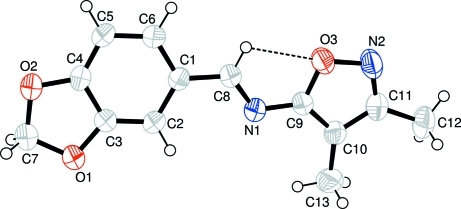
View of (I) with displacement ellipsoids drawn at the 50% probability level.

**Fig. 2.**
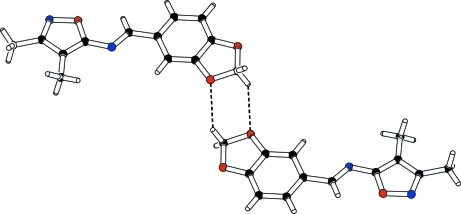
The partial packing for (I), which shows that molecules form dimers with R22(6) ring motif.

## supplementary crystallographic information

### Comment

Donor-acceptor conjugated azo-methanes are used as substrates in the preparation of a large number of bioactive and industrial compounds *via* ring closure, cycloaddition, replacement reactions, *etc* (Asiri & Khan, 2010).  The title compound (I, Fig. 1) has been prepared as a pharmaceutical intermediate.

The crystal structures of *N*-(4-chlorobenzylidene)-3,4-dimethylisoxazol -5-amine (Asiri *et al.*, 2010) and *N*-[(*E*)-1,3-benzodioxol -5-ylmethylidene]-4-methylaniline (Tahir *et al.*, 2010) have been published, which are related to (I).

In (I), the group A (C1–C8/O1/O2) of 1,3-benzodioxole-5-carbaldehyde moiety and the group B (C9—C12/N1/N2/O3) of 5-amino-3,4-dimethylisoxazole moiety are almost planar with r.m.s. deviations of 0.022 and 0.019 Å, respectively. The dihedral angle between A/B is 8.29 (3)°. The inter-molecular H-bondings of C—H···O type dimerize the molecules with *R*_2_^2^(6) ring motif (Bernstein *et al.*, 1995) (Table 1, Fig. 2). In stabilizing the molecules π–π interactions [separation: 3.7480 (13), 3.9047 (13) Å] and C—H···π interaction (Table 1) play important role in stabilizing the molecules.

### Experimental

A mixture of 1,3-benzodioxole-5-carbaldehyde (0.50 g, 3.3 mmol) and 5-amino-3,4-dimethylisoxazole (3.3 mmol) in ethanol (15 ml) was heated for 3 h. The progress of the reaction was monitored by TLC. The solid that separated from the cooled mixture was collected and recrystallized from a methanol-chloroform mixture (8:2) to give the yellow prisms of the title compound (I).

Yield: 74%; m.p. 504–505 K.

### Refinement

The H-atoms were positioned geometrically (C–H = 0.93–0.97 Å) and refined as riding with *U*_iso_(H) = x*U*_eq_(C), where x = 1.5 for methyl and x = 1.2 for aryl H-atoms.

### Crystal data

**Table:**

C_13_H_12_N_2_O_3_	*F*(000) = 512
*M**_r_* = 244.25	*D*_x_ = 1.402 Mg m^−^^3^
Monoclinic, *P*2_1_/*n*	Mo *K*α radiation, λ = 0.71073 Å
Hall symbol: -P 2yn	Cell parameters from 1447 reflections
*a* = 7.5759 (5) Å	θ = 3.4–25.3°
*b* = 10.6980 (9) Å	µ = 0.10 mm^−^^1^
*c* = 14.6307 (12) Å	*T* = 296 K
β = 102.607 (2)°	Prism, yellow
*V* = 1157.19 (16) Å^3^	0.28 × 0.24 × 0.22 mm
*Z* = 4	

### Data collection

**Table:**

Bruker Kappa APEXII CCD diffractometer	2087 independent reflections
Radiation source: fine-focus sealed tube	1447 reflections with *I* > 2σ(*I*)
graphite	*R*_int_ = 0.032
Detector resolution: 8.10 pixels mm^-1^	θ_max_ = 25.3°, θ_min_ = 3.4°
ω scans	*h* = −9→9
Absorption correction: multi-scan (*SADABS*; Bruker, 2005)	*k* = −12→12
*T*_min_ = 0.975, *T*_max_ = 0.980	*l* = −17→17
8018 measured reflections	

### Refinement

**Table:**

Refinement on *F*^2^	Primary atom site location: structure-invariant direct methods
Least-squares matrix: full	Secondary atom site location: difference Fourier map
*R*[*F*^2^ > 2σ(*F*^2^)] = 0.045	Hydrogen site location: inferred from neighbouring sites
*wR*(*F*^2^) = 0.132	H-atom parameters constrained
*S* = 1.03	*w* = 1/[σ^2^(*F*_o_^2^) + (0.0693*P*)^2^ + 0.1704*P*] where *P* = (*F*_o_^2^ + 2*F*_c_^2^)/3
2087 reflections	(Δ/σ)_max_ < 0.001
165 parameters	Δρ_max_ = 0.17 e Å^−^^3^
0 restraints	Δρ_min_ = −0.18 e Å^−^^3^

### Special details

**Table:**

Geometry. Bond distances, angles *etc*. have been calculated using the rounded fractional coordinates. All su's are estimated from the variances of the (full) variance-covariance matrix. The cell e.s.d.'s are taken into account in the estimation of distances, angles and torsion angles
Refinement. Refinement of *F*^2^ against ALL reflections. The weighted *R*-factor *wR* and goodness of fit *S* are based on *F*^2^, conventional *R*-factors *R* are based on *F*, with *F* set to zero for negative *F*^2^. The threshold expression of *F*^2^ > σ(*F*^2^) is used only for calculating *R*-factors(gt) *etc*. and is not relevant to the choice of reflections for refinement. *R*-factors based on *F*^2^ are statistically about twice as large as those based on *F*, and *R*- factors based on ALL data will be even larger.

### Fractional atomic coordinates and isotropic or equivalent isotropic displacement parameters (Å^2^)

**Table:**

	*x*	*y*	*z*	*U*_iso_*/*U*_eq_	
O1	0.6073 (2)	0.09898 (13)	0.08565 (10)	0.0729 (6)	
O2	0.6345 (2)	0.27941 (14)	0.00426 (10)	0.0702 (6)	
O3	0.6207 (2)	0.38037 (12)	0.55753 (10)	0.0699 (6)	
N1	0.6338 (2)	0.23824 (15)	0.43533 (11)	0.0487 (5)	
N2	0.6192 (3)	0.37712 (18)	0.65383 (13)	0.0742 (8)	
C1	0.6183 (2)	0.31798 (17)	0.28025 (13)	0.0457 (6)	
C2	0.6088 (3)	0.20099 (17)	0.23618 (13)	0.0493 (6)	
C3	0.6150 (3)	0.19982 (17)	0.14411 (14)	0.0490 (7)	
C4	0.6313 (3)	0.30805 (19)	0.09481 (13)	0.0510 (7)	
C5	0.6386 (3)	0.42247 (19)	0.13520 (15)	0.0618 (8)	
C6	0.6302 (3)	0.42546 (18)	0.22912 (14)	0.0572 (7)	
C7	0.6271 (3)	0.1475 (2)	−0.00210 (15)	0.0687 (8)	
C8	0.6190 (2)	0.33074 (18)	0.37881 (13)	0.0497 (7)	
C9	0.6331 (3)	0.26031 (18)	0.52782 (13)	0.0478 (7)	
C10	0.6405 (2)	0.18039 (18)	0.60027 (13)	0.0477 (7)	
C11	0.6292 (3)	0.2593 (2)	0.67649 (14)	0.0556 (7)	
C12	0.6270 (3)	0.2203 (2)	0.77363 (15)	0.0716 (9)	
C13	0.6585 (3)	0.0425 (2)	0.60257 (15)	0.0697 (9)	
H2	0.59873	0.12747	0.26857	0.0591*	
H5	0.64865	0.49515	0.10183	0.0741*	
H6	0.63264	0.50254	0.25877	0.0686*	
H7A	0.73721	0.11561	−0.01717	0.0824*	
H7B	0.52555	0.12203	−0.05118	0.0824*	
H8	0.60811	0.41048	0.40229	0.0596*	
H12A	0.51285	0.18213	0.77471	0.1074*	
H12B	0.64420	0.29227	0.81383	0.1074*	
H12C	0.72268	0.16145	0.79518	0.1074*	
H13A	0.69485	0.01447	0.54709	0.1045*	
H13B	0.54443	0.00536	0.60531	0.1045*	
H13C	0.74801	0.01816	0.65679	0.1045*	

### Atomic displacement parameters (Å^2^)

**Table:**

	*U*^11^	*U*^22^	*U*^33^	*U*^12^	*U*^13^	*U*^23^
O1	0.1287 (13)	0.0486 (9)	0.0459 (9)	−0.0125 (8)	0.0292 (9)	−0.0072 (7)
O2	0.1149 (12)	0.0587 (10)	0.0411 (8)	−0.0105 (8)	0.0259 (8)	−0.0007 (7)
O3	0.1182 (12)	0.0479 (9)	0.0477 (9)	0.0126 (8)	0.0273 (8)	−0.0003 (7)
N1	0.0606 (10)	0.0462 (9)	0.0407 (9)	−0.0010 (7)	0.0143 (7)	0.0013 (7)
N2	0.1179 (16)	0.0650 (13)	0.0447 (11)	0.0151 (10)	0.0288 (10)	−0.0032 (9)
C1	0.0549 (11)	0.0427 (10)	0.0424 (10)	0.0009 (8)	0.0170 (8)	0.0031 (8)
C2	0.0648 (12)	0.0409 (10)	0.0445 (11)	−0.0023 (9)	0.0172 (9)	0.0050 (9)
C3	0.0631 (12)	0.0423 (11)	0.0432 (11)	−0.0035 (9)	0.0148 (9)	−0.0020 (9)
C4	0.0652 (12)	0.0526 (12)	0.0373 (10)	−0.0033 (9)	0.0157 (9)	0.0023 (9)
C5	0.0975 (16)	0.0443 (12)	0.0495 (12)	−0.0033 (10)	0.0291 (11)	0.0083 (9)
C6	0.0860 (15)	0.0407 (11)	0.0497 (12)	−0.0020 (10)	0.0253 (10)	−0.0009 (9)
C7	0.1030 (17)	0.0597 (14)	0.0456 (12)	−0.0082 (12)	0.0210 (11)	−0.0036 (10)
C8	0.0656 (13)	0.0431 (10)	0.0434 (11)	−0.0004 (9)	0.0186 (9)	−0.0022 (9)
C9	0.0591 (12)	0.0446 (11)	0.0410 (11)	0.0003 (8)	0.0140 (9)	−0.0023 (9)
C10	0.0517 (11)	0.0505 (12)	0.0404 (11)	−0.0020 (8)	0.0091 (8)	0.0009 (9)
C11	0.0582 (12)	0.0666 (14)	0.0426 (12)	0.0025 (10)	0.0122 (9)	0.0005 (10)
C12	0.0825 (16)	0.0934 (18)	0.0410 (12)	−0.0012 (13)	0.0179 (11)	0.0039 (12)
C13	0.0987 (17)	0.0543 (13)	0.0523 (14)	−0.0088 (11)	0.0083 (12)	0.0070 (10)

### Geometric parameters (Å, °)

**Table:**

O1—C3	1.370 (2)	C9—C10	1.353 (3)
O1—C7	1.423 (3)	C10—C11	1.416 (3)
O2—C4	1.365 (2)	C10—C13	1.481 (3)
O2—C7	1.415 (3)	C11—C12	1.485 (3)
O3—N2	1.412 (2)	C2—H2	0.9300
O3—C9	1.366 (2)	C5—H5	0.9300
N1—C8	1.279 (2)	C6—H6	0.9300
N1—C9	1.375 (2)	C7—H7A	0.9700
N2—C11	1.301 (3)	C7—H7B	0.9700
C1—C2	1.403 (3)	C8—H8	0.9300
C1—C6	1.385 (3)	C12—H12A	0.9600
C1—C8	1.447 (3)	C12—H12B	0.9600
C2—C3	1.358 (3)	C12—H12C	0.9600
C3—C4	1.384 (3)	C13—H13A	0.9600
C4—C5	1.355 (3)	C13—H13B	0.9600
C5—C6	1.390 (3)	C13—H13C	0.9600
			
C3—O1—C7	106.12 (15)	C10—C11—C12	126.98 (19)
C4—O2—C7	106.20 (16)	C1—C2—H2	121.00
N2—O3—C9	108.09 (14)	C3—C2—H2	121.00
C8—N1—C9	119.01 (17)	C4—C5—H5	122.00
O3—N2—C11	105.39 (17)	C6—C5—H5	122.00
C2—C1—C6	119.69 (17)	C1—C6—H6	119.00
C2—C1—C8	122.06 (17)	C5—C6—H6	119.00
C6—C1—C8	118.24 (17)	O1—C7—H7A	110.00
C1—C2—C3	117.05 (17)	O1—C7—H7B	110.00
O1—C3—C2	128.35 (17)	O2—C7—H7A	110.00
O1—C3—C4	109.26 (17)	O2—C7—H7B	110.00
C2—C3—C4	122.39 (18)	H7A—C7—H7B	108.00
O2—C4—C3	109.91 (17)	N1—C8—H8	118.00
O2—C4—C5	128.19 (19)	C1—C8—H8	118.00
C3—C4—C5	121.89 (18)	C11—C12—H12A	109.00
C4—C5—C6	116.46 (19)	C11—C12—H12B	109.00
C1—C6—C5	122.49 (18)	C11—C12—H12C	109.00
O1—C7—O2	108.37 (16)	H12A—C12—H12B	109.00
N1—C8—C1	123.51 (17)	H12A—C12—H12C	110.00
O3—C9—N1	119.35 (16)	H12B—C12—H12C	109.00
O3—C9—C10	109.80 (16)	C10—C13—H13A	109.00
N1—C9—C10	130.84 (18)	C10—C13—H13B	110.00
C9—C10—C11	103.94 (17)	C10—C13—H13C	109.00
C9—C10—C13	129.41 (18)	H13A—C13—H13B	109.00
C11—C10—C13	126.65 (18)	H13A—C13—H13C	109.00
N2—C11—C10	112.77 (18)	H13B—C13—H13C	109.00
N2—C11—C12	120.26 (19)		
			
C7—O1—C3—C2	−177.5 (2)	C6—C1—C8—N1	−170.00 (18)
C7—O1—C3—C4	2.4 (2)	C1—C2—C3—O1	−179.6 (2)
C3—O1—C7—O2	−3.8 (2)	C1—C2—C3—C4	0.5 (3)
C7—O2—C4—C3	−2.4 (2)	O1—C3—C4—O2	0.0 (3)
C7—O2—C4—C5	178.9 (2)	O1—C3—C4—C5	178.8 (2)
C4—O2—C7—O1	3.8 (2)	C2—C3—C4—O2	179.9 (2)
C9—O3—N2—C11	−0.4 (2)	C2—C3—C4—C5	−1.3 (4)
N2—O3—C9—N1	178.87 (19)	O2—C4—C5—C6	179.1 (2)
N2—O3—C9—C10	−0.3 (2)	C3—C4—C5—C6	0.5 (3)
C9—N1—C8—C1	179.97 (18)	C4—C5—C6—C1	1.1 (3)
C8—N1—C9—O3	−1.4 (3)	O3—C9—C10—C11	0.8 (2)
C8—N1—C9—C10	177.5 (2)	O3—C9—C10—C13	−178.49 (18)
O3—N2—C11—C10	0.9 (3)	N1—C9—C10—C11	−178.2 (2)
O3—N2—C11—C12	−178.74 (19)	N1—C9—C10—C13	2.5 (4)
C6—C1—C2—C3	1.1 (3)	C9—C10—C11—N2	−1.1 (3)
C8—C1—C2—C3	−177.91 (18)	C9—C10—C11—C12	178.5 (2)
C2—C1—C6—C5	−2.0 (3)	C13—C10—C11—N2	178.2 (2)
C8—C1—C6—C5	177.09 (19)	C13—C10—C11—C12	−2.2 (3)
C2—C1—C8—N1	9.0 (3)		

### Hydrogen-bond geometry (Å, °)

**Table:**

Cg1 is the centroid of the C1–C6 benzene ring.

**Table:**

*D*—H···*A*	*D*—H	H···*A*	*D*···*A*	*D*—H···*A*
C7—H7B···O1^i^	0.97	2.58	3.264 (3)	128
C12—H12A···Cg1^ii^	0.96	2.95	3.763 (2)	143

Symmetry codes: (i) −*x*+1, −*y*, −*z*; (ii) *x*−1/2, −*y*+1/2, *z*+1/2.

## References

[bb1] Asiri, A. M. & Khan, S. A. (2010). *Molecules*, **15**, 6850–6858. 10.3390/molecules15106850 PMC6259180 20938399

[bb2] Asiri, A. M., Khan, S. A. & Tahir, M. N. (2010). *Acta Cryst.* E**66**, o2127. 10.1107/S1600536810029284 PMC3007510 21588416

[bb3] Bernstein, J., Davis, R. E., Shimoni, L. & Chang, N.-L. (1995). *Angew. Chem. Int. Ed. Engl.* **34**, 1555–1573.

[bb4] Bruker (2005). *SADABS* Bruker AXS Inc., Madison, Wisconsin, USA.

[bb5] Bruker (2009). *APEX2* and *SAINT* Bruker AXS Inc., Madison, Wisconsin, USA.

[bb6] Farrugia, L. J. (1997). *J. Appl. Cryst.* **30**, 565.

[bb7] Farrugia, L. J. (1999). *J. Appl. Cryst.* **32**, 837–838.

[bb8] Sheldrick, G. M. (2008). *Acta Cryst.* A**64**, 112–122. 10.1107/S0108767307043930 18156677

[bb9] Spek, A. L. (2009). *Acta Cryst.* D**65**, 148–155. 10.1107/S090744490804362X PMC2631630 19171970

[bb10] Tahir, M. N., Shad, H. A., Khan, M. N. & Tariq, R. H. (2010). *Acta Cryst.* E**66**, o3293. 10.1107/S1600536810048038 PMC3011519 21589572

